# Supplementary material for: Transplanting FVIII/ET3-secreting cells in fetal sheep increases FVIII levels long-term without inducing immunity or toxicity
Source: Nat Commun. 2023 Jul 14;14:4206. doi: 10.1038/s41467-023-39986-1 (PMC10349136; doi:10.1038/s41467-023-39986-1)
Supplement: Supplementary file 1 — Supplementary Information [file 41467_2023_39986_MOESM1_ESM.pdf]

Transplanting FVIII/ET3-Secreting Cells in Fetal Sheep Increases FVIII Levels Long-Term Without Inducing Immunity or Toxicity

SUPPLEMENTARY TABLE 1

Study Design and Outcomes

ANIMAL ID	CELL DOSE/ KG *	VCN	IU FVIII 10^6 CELLS/ 24H	CELL DOSE USING WEIGHT @ BIRTH	YEARS POST-IUTX
LONG-TERM EVALUATION					END-POINTS or OUTCOMES
17010 (M)	10^7	0.35	8.4	2.19 x10^5	5
17011 (F)	10^7	0.35	8.4	2.12 x10^5	HA Carrier&
17013 (F)	10^7	0.35	8.4	3.33 x10^5	HA Carrier&
17019 (M)	10^8	0.35	8.4	2.70 x10^6	Continued Evaluation&&
17020 (M)	10^8	0.35	8.4	2.43 x10^6	4.5
17021 (F)	10^8	0.35	8.4	4.55 x10^6	4.5
18007 (F)	10^8	0.4	9.7	1.92 x10^6	HA Carrier&
18009 (M)	10^8	0.4	9.7	5.52 x10^6	3.5
19001 (F)	10^8	0.5	5	2.50 x10^6	HA Carrier&
19006 (M)	10^7	4.7	32	3.70 x10^5	Continued Evaluation&&
19007 (F)	10^7	4.7	32	3.45 x10^5	2.33
19008 (M)	10^7	4.7	32	2.78 x10^5	Continued Evaluation&&
19011 (M)	4x10^8	4.7	32	1.00 x10^7	2.33
SHORT-TERM EVALUATION				≈ MONTHS POST-IUTX	
18001 (M)	10^8	0.35	8.4	3	
18002 (F)	10^8	0.35	8.4	3	
18008 (F)	10^8	0.4	9.7	3	
1002**HA	5x10^7	0.5	5	3	
LOST TO ANALYSIS				CAUSE	
USU1**HA	10^8	0.5	5	Late-term abortion as reported in cloned animals	
USU2**HA	10^8	0.5	5		
USU3**HA	10^8	0.5	5		
17012 (F)	10^7	0.35	8.4	Umbilical Torsion	
17018(M)	10^8	0.35	8.4	Stillborn/low weight	
17022 (M)	10^8	0.35	8.4	Pneumonia	
19009 (M)	10^8	4.7	32	Infection	
19012 (M)	4x10^8	4.7	32	Pneumonia	

* Estimated fetal weight at time of injection 100g

** Hemophilia A animal, males

& HA Carriers

&& Will continue to be evaluated

F Female

M male

Supplementary Table 2: Summary of upregulated endothelial- and hepatocyte-specific genes in Liver of IUTx Recipients

			Short-Term Evaluation				Long-Term Evaluation					
**Liver Endothelial Specific Genes**												
Name	Ensembl ID	Protein	1002	18001	18002	18008	17010	17020	17021	18009	19007	19011
PROX1	ENSG00000117707	Prospero homeobox 1	+	+	+	+	+	+	+	+	+	+
CD36	ENSG00000135218	Platelet glycoprotein IV		+	+	+	+	+	+	+	+	
MRC1	ENSG00000260314	Mannose receptor C-type 1	+	+	+	+		+	+	+	+	+
LYVE1	ENSG00000133800	Lymphatic vessel endothelial hyaluronan receptor 1	+	+	+	+			+			
VWF	ENSG00000110799	von Willebrand Factor		+					+			+
F8	ENSG00000185010	Coagulation Factor VIII	+	+	+	+		+	+	+	+	+
MRC2	ENSG00000011028	Mannose receptor C type 2										
PECAM1	ENSG00000261371	PECAM 1 / CD31		+					+			
ICAM1	ENSG00000090339	Intercellular adhesion molecule 1										
**Hepatocyte Specific Genes**												
F9	ENSG00000101981	Coagulation Factor IX	+	+	+	+		+	+	+	+	+
MASP2	ENSG00000009724	MBL associated serine protease 2		+				+	+	+		+
LEPR	ENSG00000116678	Leptin receptor	+	+	+	+			+	+		+
CRP	ENSG00000132693	C-reactive protein		+				+	+	+		+
APOA2	ENSG00000158874	Apolipoprotein A2						+		+		+
F5	ENSG00000198734	Coagulation Factor V	+	+	+	+	+	+	+	+	+	+
PROX1	ENSG00000117707	Prospero homeobox 1	+	+	+	+	+	+	+	+	+	+
APOB	ENSG00000084674	Apolipoprotein B		+	+			+	+	+		+
CP	ENSG00000047457	Ceruloplasmin							+	+		+
ALB	ENSG00000163631	Albumin		+	+			+	+	+	+	+
AFP	ENSG00000081051	Alpha-fetoprotein		+	+							
FGB	ENSG00000171564	Fibrinogen beta chain	+	+	+		+	+	+	+		+
FGA	ENSG00000171560	Fibrinogen alpha chain	+	+	+			+	+	+	+	+
FGG	ENSG00000171557	Fibrinogen gamma chain	+	+	+			+	+	+	+	+
APOM	ENSG00000204444	Apolipoprotein M		+	+			+	+	+	+	+
GSTA2	ENSG00000244067	Glutathione S-transferase alpha 2						+	+	+	+	+
CYP3A7	ENSG00000160870	Cytochrome P450 family 3 subfamily A member 7								+	+	
CYP3A4	ENSG00000160868	Cytochrome P450 family 3 subfamily A member 4							+	+		
TFR2	ENSG00000106327	Transferrin receptor 2	+		+	+		+	+	+	+	+
FGL1	ENSG00000104760	Fibrinogen like 1					+	+	+	+	+	
CYP7A1	ENSG00000167910	Cytochrome P450 family 7 subfamily A member 1								+	+	+
HHEX	ENSG00000152804	Hematopoietically expressed homeobox				+			+	+		+
CYP2C9	ENSG00000138109	Cytochrome P450 family 2 subfamily C member 9						+	+	+	+	+
CYP2C8	ENSG00000138115	Cytochrome P450 family 2 subfamily C member 8						+	+	+		+
CYP2E1	ENSG00000130649	Cytochrome P450 family 2 subfamily E member 1					+	+	+	+	+	+
LRP5	ENSG00000162337	LDL receptor related protein 5								+		+
SERPINH1	ENSG00000149257	Serpin family H member 1										
APOA1	ENSG00000118137	Apolipoprotein A1	+	+	+	+		+	+	+	+	+
SERPINA10	ENSG00000140093	Serpin family A member 10		+	+	+		+	+	+		+
SERPINA6	ENSG00000170099	Serpin family A member 6			+							+
AQP9	ENSG00000103569	Aquaporin 9								+	+	+
CYP1A1	ENSG00000140465	Cytochrome P450 family 1 subfamily A member 1						+	+	+		+
CYP1A2	ENSG00000140505	Cytochrome P450 family 1 subfamily A member 2					+	+	+	+	+	+
TAT	ENSG00000198650	Tyrosine aminotransferase	+	+	+	+	+	+	+	+	+	+
ASGR1	ENSG00000141505	Asialoglycoprotein receptor 1		+	+	+			+	+		+
APOH	ENSG00000091583	Apolipoprotein H		+				+	+	+	+	+
TTR	ENSG00000118271	Transthyretin								+	+	+
CEBPA	ENSG00000245848	CCAAT enhancer binding protein alpha										+
CYP2A6	ENSG00000255974	Cytochrome P450 family 2 subfamily A member 6						+	+	+	+	+
CYP2A7	ENSG00000198077	Cytochrome P450 family 2 subfamily A member 7						+		+		
CYP2B6	ENSG00000197408	Cytochrome P450 family 2 subfamily B member 6						+	+		+	+
HNF4A	ENSG00000101076	Hepatocyte nuclear factor 4 alpha	+	+	+	+	+	+	+	+	+	+
PCK1	ENSG00000124253	Phosphoenolpyruvate carboxykinase 1				+	+	+	+	+	+	+
CYP2D6	ENSG00000100197	Cytochrome P450 family 2 subfamily D member 6						+	+	+	+	+
OTC	ENSG00000036473	Ornithine transcarbamylase	+	+	+			+	+	+	+	+
CPS1	ENSG00000021826	Urea cycle enzyme CPS1.	+	+	+			+	+	+	+	+

Supplementary Table 3: Summary of upregulated genes in Lung of IUTx Recipients

			Short-Term Evaluation				Long-Term Evaluation					
Gene Name	Ensembl ID	Protein	1002	18001	18002	18008	17010	17020	17021	18009	19007	19011
AQP1	ENSG00000240583	Aquaporin 1		+			+	+		+	+	+
AQP3	ENSG00000165272	Aquaporin 3										
AQP4	ENSG00000171885	Aquaporin 4	+				+	+		+		
AQP5	ENSG00000161798	Aquaporin 5					+			+	+	
CLDN1	ENSG00000163347	Claudin 1			+		+			+		
CLDN3	ENSG00000165215	Claudin 3		+	+							+
CLDN4	ENSG00000189143	Claudin 4										
CLDN7	ENSG00000181885	Claudin 7		+	+		+				+	+
CLDN18	ENSG00000066405	Claudin 18	+	+	+		+	+		+	+	+
SCNN1B	ENSG00000168447	Sodium channel epithelial 1 subunit beta	+	+						+	+	
ATP1A1	ENSG00000163399	ATPase Na+/K+ transporting subunit alpha 1	+	+			+	+		+	+	+
SFTPC	ENSG00000168484	Surfactant protein C	+				+	+			+	+
ABCA3	ENSG00000167972	ATP binding cassette subfamily A member 3	+				+	+			+	+
CAV1	ENSG00000105974	Caveolin 1										
CDH1	ENSG00000039068	Cadherin 1	+	+	+	+	+	+	+	+	+	+
CDH2	ENSG00000170558	Cadherin 2										
gp36	ENSG00000169223	lectin, mannose binding 2	+	+			+			+	+	+
AGER	ENSG00000204305	receptor specific for advanced glycosylation end-products	+	+			+	+		+	+	+
CFTR	ENSG00000001626	CF transmembrane conductance regulator		+		+	+	+			+	

Supplementary Table 4: Summary of upregulated genes in Thymus of IUTx Recipients

			Short-Term Evaluation				Long-Term Evaluation				
Name	Ensembl ID	Protein	1002	18001	18002	18008	17010	17020	18009	19007	19011
KRT5	ENSG00000186081	Cytokeratin 5		+				+			+
KRT8	ENSG00000170421	Cytokeratin 8									
KRT13	ENSG00000171401	Cytokeratin 13									
F8	ENSG00000185010	Coagulation Factor VIII						+			+
AIRE	ENSG00000160224	AIRE									
PDPN	ENSG00000162493	Podoplanin									
CD200/MOX1/MOX2	ENSG00000091972	Immunoregulatory interactions between Lymphoid & non-Lymphoid cells						+			
COL4A3	ENSG00000169031	Collagen type IV alpha 3 chain									
EpCAM	ENSG00000119888	epithelial cell adhesion molecule									+
FOXN1	ENSG00000109101	Forkhead box N1						+			+
ASCL1	ENSG00000139352	Achaete-scute family bHLH transcription factor 1									
INSM1	ENSG00000173404	INSM transcriptional repressor 1									
EHF	ENSG00000135373	ETS homologous factor									
POU2F3	ENSG00000137709	POU domain, class 2, transcription factor 3									
SPIB	ENSG00000269404	Spi-B transcription factor									
CLDN3	ENSG00000165215	Claudin3									
CLDN4	ENSG00000189143	Claudin 4									

Supplementary Table 5: Summary of upregulated oocyte-specific genes in Ovary of IUTx Recipients

Name	Ensembl ID	Protein	17021	18009	19007
GDF9	ENSG00000164404	growth differentiation factor 9			
MLF1P/CENPU	ENSG00000151725	MLF1-interacting protein			
PAIP1	ENSG00000172239	polyadenylate-binding protein-interacting protein 1			
BTG4	ENSG00000137707	B-cell translocation gene 4			
NPM2	ENSG00000158806	nucleoplasmin 2			
c-mos	ENSG00000172680	c-mos proto-oncogene			
ZP3	ENSG00000188372	zona pellucida glycoprotein 3			
ZP2	ENSG00000103310	zona pellucida glycoprotein 2			
ZP1	ENSG00000149506	zona pellucida glycoprotein 1			
BMP15	ENSG00000130385	Bone morphogenetic protein 15			
ZAR1	ENSG00000182223	Zygote arrest 1			

Supplementary Table 6: Summary of upregulated sperm-specific genes in Testicles of IUTx Recipients

Name	Ensembl ID	Protein	17010	17020	19011
PRM1	ENSG00000175646	Sperm Protamine P1	+	+	
PRM2	ENSG00000122304	Sperm Protamine P2			
TNP2	ENSG00000178279	Transition Protein 2	+	+	
TSSK6	ENSG00000178093	Testis specific serine kinase 6	+	+	
DNAJC4	ENSG00000110011	DnaJ heat shock protein family (Hsp40) member C4			
NUPR2	ENSG00000185290	Nuclear protein 2, transcriptional regulator			
CRISP2	ENSG00000124490	Cysteine rich secretory protein 2	+	+	
SMCP	ENSG00000163206	Sperm mitochondria associated cysteine rich protein			

Supplementary Table 7

Antibody	Application Tested	Vendor	Catalog Number	Clone	Dilution	Lot	Validation for Species and Application	In-house Validation
Rabbit Anti-Human Ku80 Antibody	IHC-IF	Cell Signaling Technologies	2753S	C48E7	1::100	2	https://www.cellsignal.com/datasheet.jsp?productId=2753&images=1	The antibody was validated to not cross-react with sheep targets by using non-transplanted sheep control tissues (n=>3)
Mouse Anti-human FVIII Antibody	IHC-IF	Sekisui Diagnostics (Biomedica)	ESH-8	C2-2248:2285	1::200	151210	https://www.invitech.co.uk/index.php?route=pavblog/blog&id=43	The antibody was validated to not cross-react with sheep targets by testing in a Hemophilic animal known NOT to express sheep FVIII, as well as by using non-transplanted sheep control tissues (n=>3)
Rabbit Anti-Human Ku80 Antibody	IHC-P	Cell Signaling Technologies	2180	C48E7	1::100	1	https://www.cellsignal.com/datasheet.jsp?productId=2180&images=1	The antibody was validated to not cross-react with sheep targets by using non-transplanted sheep control tissues (n=>3)
Mouse Anti-HepPar1 Antibody	IHC-P	NeoBiotechnologies	MSM4-966-P1	HepPar1	1::400	966P221018	https://www.neobiotechnologies.com/pdf/heppar1-antibody-4-966	The antibody was validated to not cross-react with sheep HepPar1 by using non-transplanted sheep control tissues (n=>3)
Rabbit Anti-LYVE1 Antibody	IHC-P	AbCam	Ab219556	EPR21857	1::1000	GR3340292-11	https://www.abcam.com/products/primary-antibodies/lyve1-antibody-epr21857-ab219556.html	The antibody was validated to not cross-react with sheep LYVE1 by using non-transplanted sheep control tissues (n=>3)
Mouse Anti-Human Albumin Antibody	IHC-P	Invitrogen	MA1-19174	AL-01	1::200	XI3705305	https://www.thermofisher.com/order/genome-database/dataSheetPdf?producttype=antibody&productsubtype=antibody_primary&productId=MA1-19174&version=288	The antibody was validated to not cross-react with sheep albumin by using non-transplanted sheep control tissues (n=>3)
Anti-Sheep IFNg Antibody	ELISpot	MabTech	3119-4APW-2	MT17.1	1::2000	B23	https://www.mabtech.com/products/elispot-plus-bovine-ifn-g-alp-3119-4apw	None Needed
Anti-Sheep IL4 antibody	ELISpot	MabTech	3118-2A	bIL4-II	1::1000	B7	https://www.mabtech.com/products/elispot-flex-bovine-il-4-alp-3118-2a-0	None Needed
Donkey Anti-Sheep IgG:Alkaline Phosphatase	Anti-ET3 IgG ELISA	Bio-Rad	STAR88A	Polyclonal	1::1000	146177	https://images.bio-rad-antibodies.com/datasheets/datasheet-STAR88A.pdf	None Needed
Rabbit Anti-Sheep IgM H&L ALP	Anti-ET3 IgM ELISA	Abcam	ab112761	Polyclonal	1::1000	GR3282345-1	https://www.abcam.com/products/secondary-antibodies/rabbit-sheep-igm-hl-alkaline-phosphatase-ab112761.html	None Needed

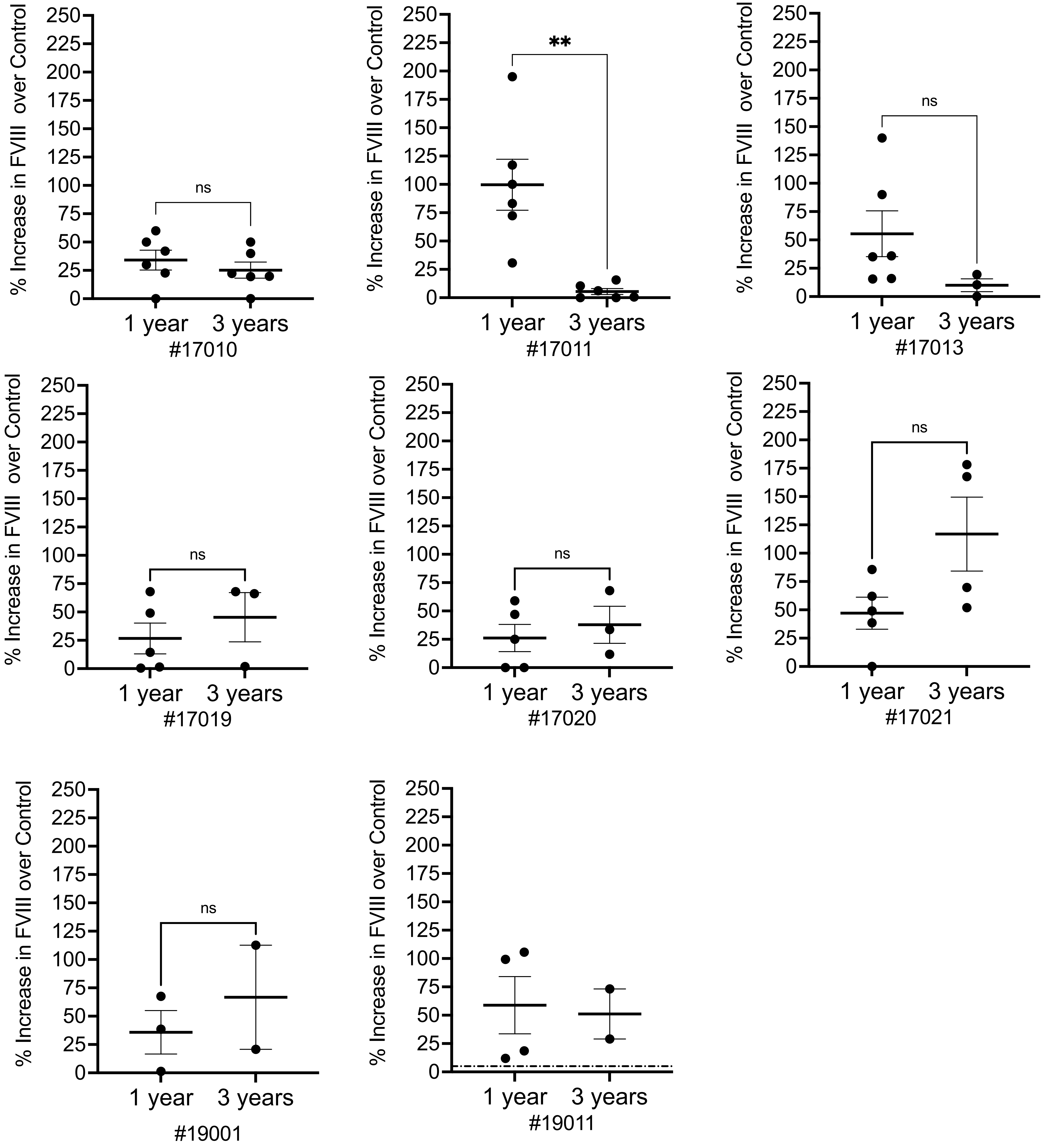
% Increase in FVIII over Control
250
225
200
175
150
125
100
75
50
25
0
1 year
3 years
ns
#17010
**
#17011
ns
#17013
ns
#17019
ns
#17020
ns
#17021
ns
#19001
#19011

Supplementary Figure 1. *Therapeutic effect did not decline significantly with time after transplant.* Analysis of the differences between mean FVIII activity during the first and the third year after transplant (n=2 to n=6 dependent on animal, with each dot representing a different sample collected and tested at a different time point). All but one recipient maintained (or did not change significantly) levels of FVIII activity despite the steady increase in weight. Dotted line corresponds to 5% FVIII activity. Data are shown as mean of FVIII activity at different blood collections ± SEM. Two-tailed paired Student's t-test were performed to determine if there were significant differences in mean FVIII activity levels between year 1 and year 3. $p<0.05$ was considered significant and **p=0.0088. p values for each one of the animals are indicated in parentheses: 17010(p=0.3668); 17013(p=0.0865); 17019(p=0.8928); 17020(p=0.7957); 17021(p=0.2332); 19001 (p=0.74); 19011(p=0.2243).

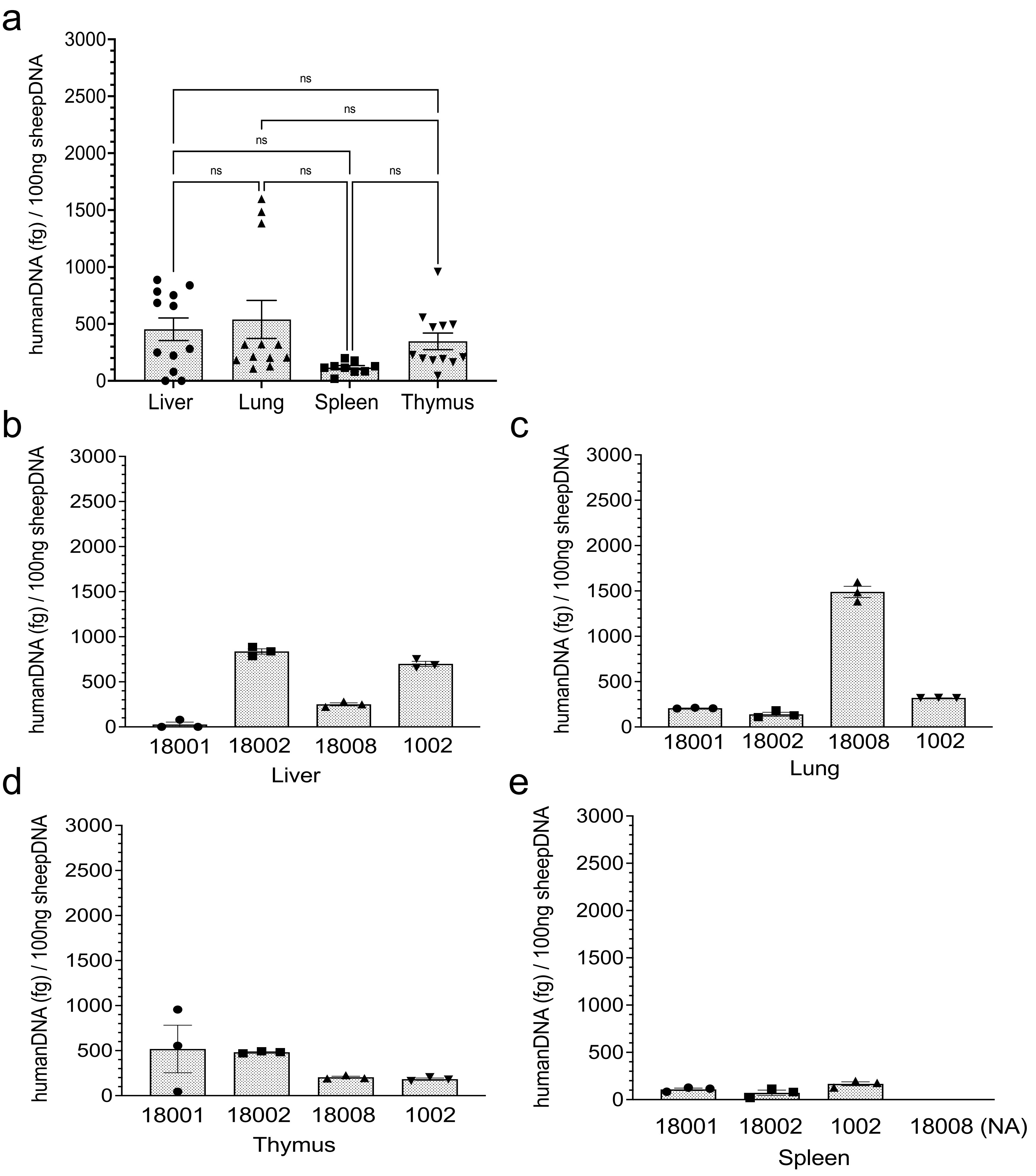
a
b
c
d
e
humanDNA (fg) / 100ng sheepDNA
3000
2500
2000
1500
1000
500
0
ns
Liver
Lung
Spleen
Thymus
18001
18002
18008
1002
18008 (NA)

Supplementary Figure 2. *Human genomic DNA can be found in different tissues after IUTx.* a) qPCR was performed in triplicate (n=4 animals) using human specific ALU primers on DNA isolated from liver, lung, spleen, and thymus, and the amount of human genomic was extrapolated using a standard curve prepared with DNA isolated from amounts of human and sheep DNA, every sample was run in triplicate; b-e) the amount of human DNA in specific tissues of the different animals. Data are shown as Mean ± SEM. One-way ANOVA followed by Tukey's multiple comparison test was used to determine significant differences and $p \leq 0.05$ was considered significant. (ns >0.05) and ns p values are detailed in sequence from left to right: 0.9539; 0.4679; 0.9184; 0.2338; 0.6571; 0.8095.

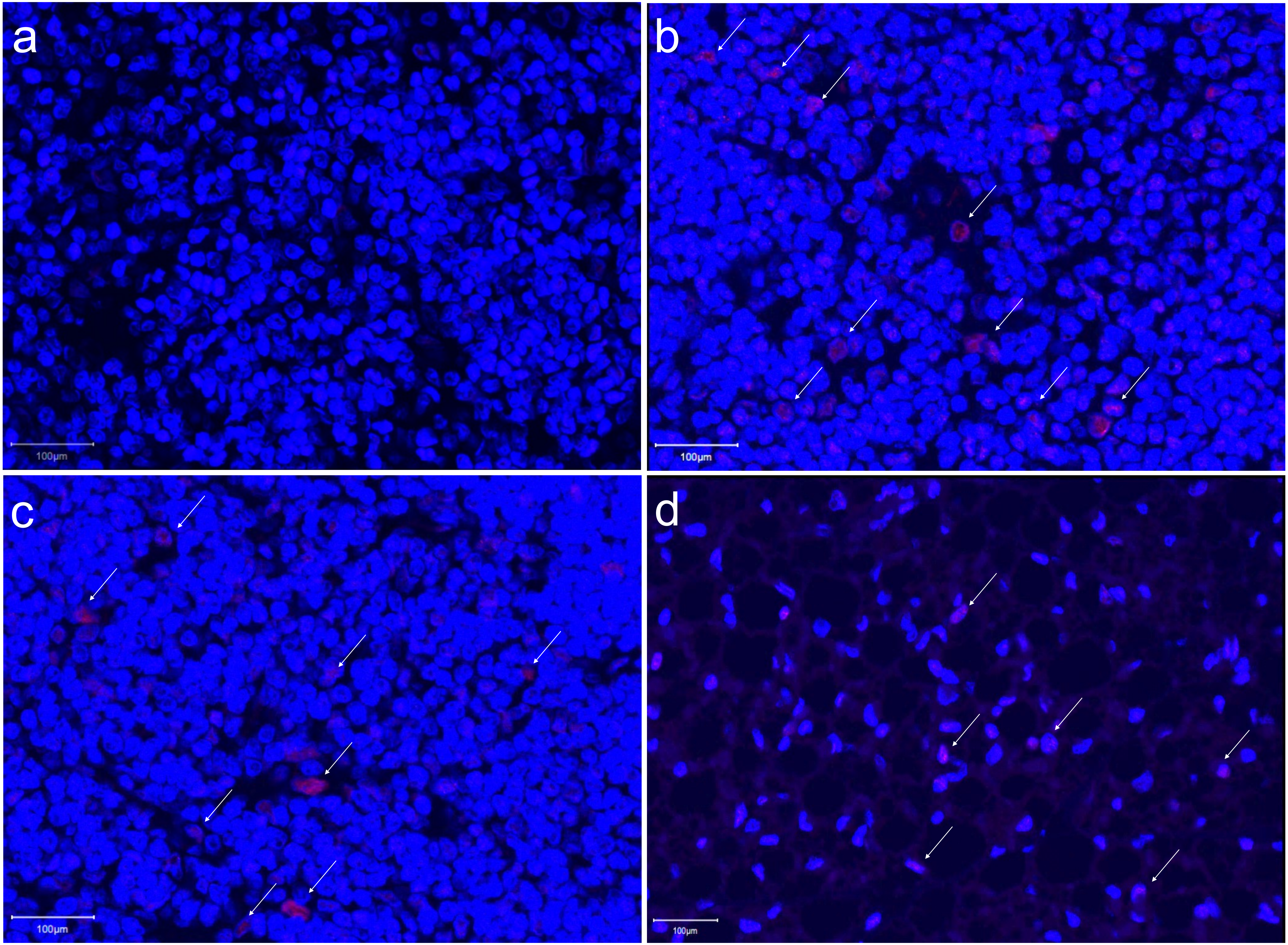
a
100μm
b
100μm
c
100μm
d
100μm

Supplementary Figure 3. *Human PLC-mcoET3 are present in the thymus of IUTx animals.* Representative images of Ku80 staining of thymic tissue sections of (a) control non-transplanted animal, (b-d) IUTx recipients (n=3 experiments). Confocal images were acquired with an Olympus Fluoview FV1000 confocal microscope with an Olympus UPlanFLN- 40x/1.30 oil objective; white arrows denote positive nuclear KU80 staining. White arrows indicate examples of positive cells.

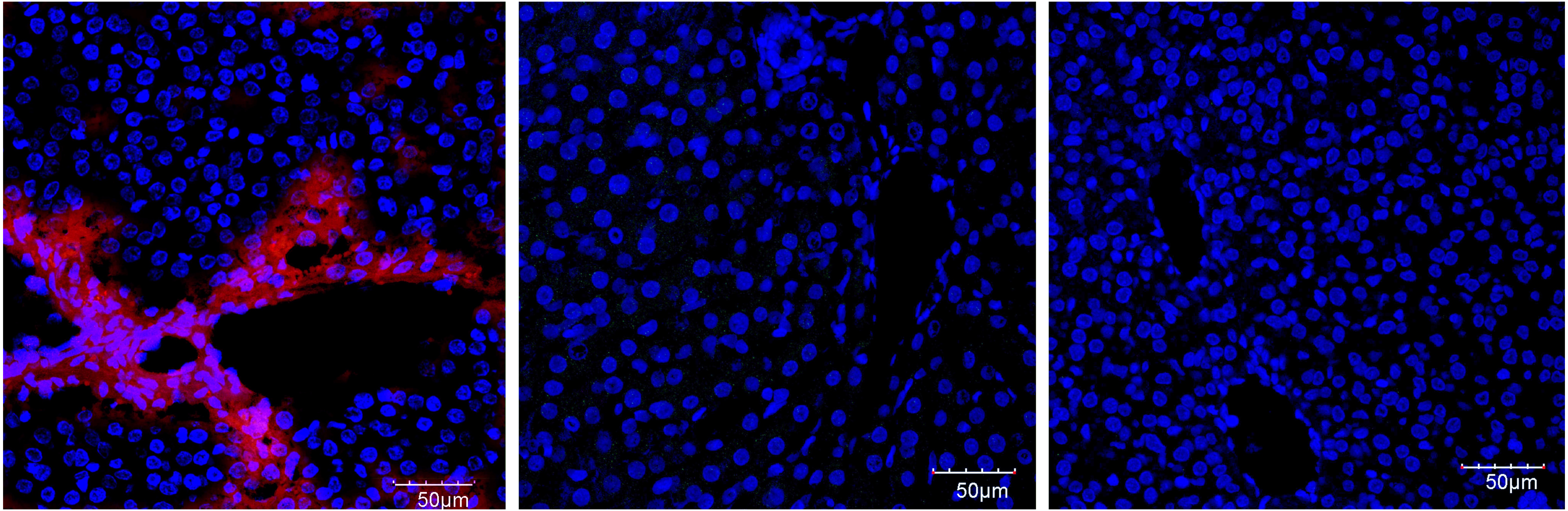
50μm
50μm
50μm

Supplementary Figure 4. *Hemophilia A sheep are cross-reactive material negative***.** Sheep with HA do not produce factor VIII protein (cross-reactive material negative). Representative images of FVIII staining of liver tissue (a) from wild type control sheep and (b and c) from archived slides of 2 non-transplanted HA animals (n=2 experiments). Images were acquired and processed as above.

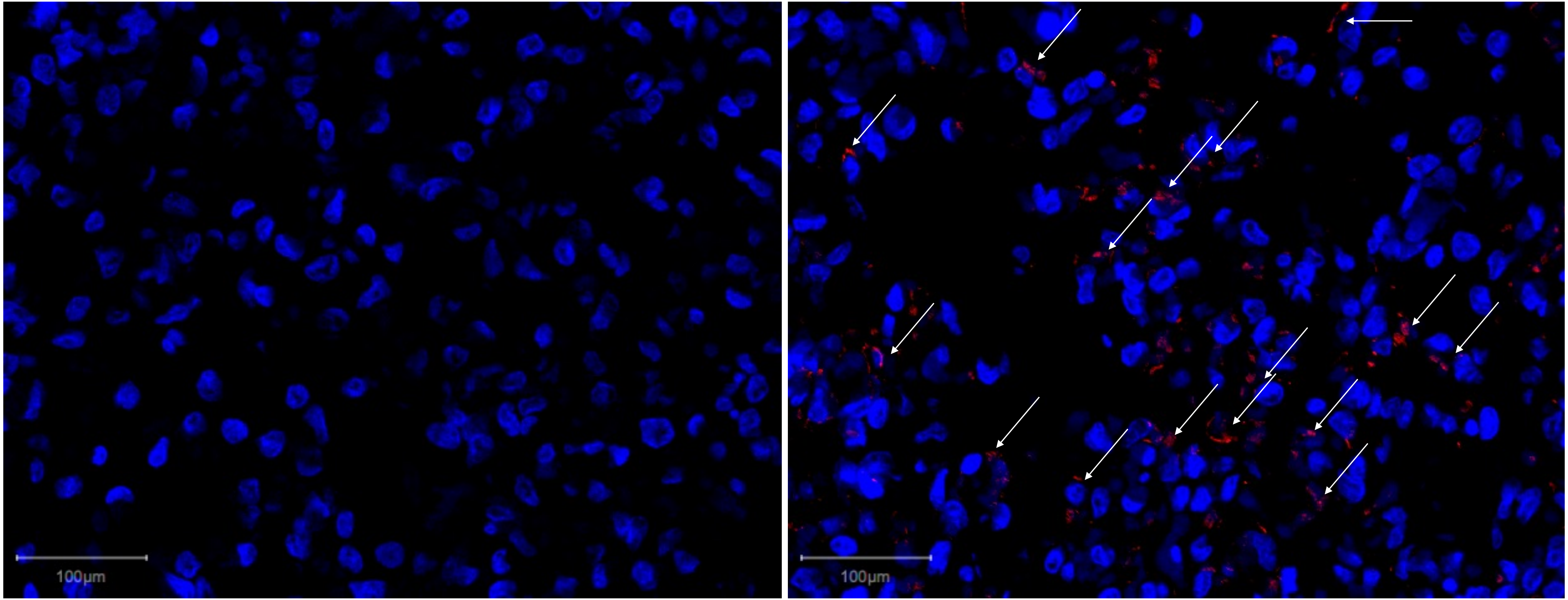
100µm
100µm

Supplementary Figure 5. *Human PLC-mcoET3 are present in the thymus of IUTx HA animal and express FVIII.* Representative images of FVIII staining of thymic tissue sections of a HA animal; (a) isotype control, (b) FVIII staining. Images were acquired and processed as above. White arrows indicate examples of positive cells (n=3 experiments).

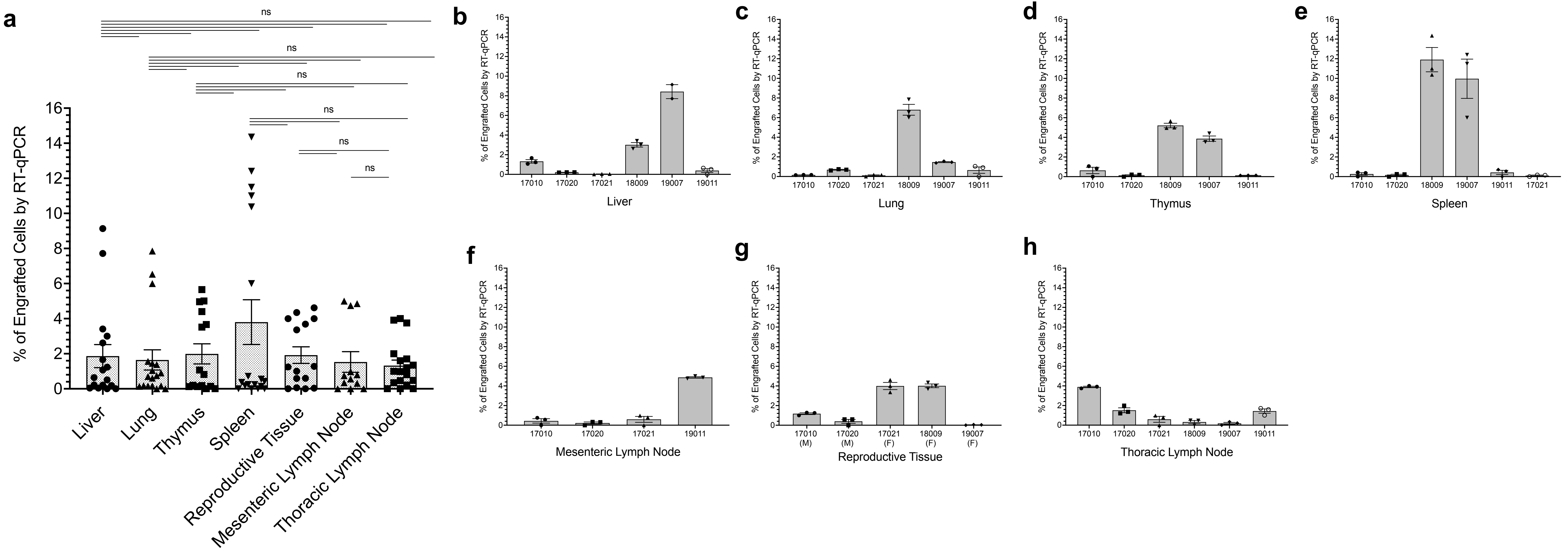

a
b
c
d
e
f
g
h
% of Engrafted Cells by RT-qPCR
ns
Liver
Lung
Thymus
Spleen
Reproductive Tissue
Mesenteric Lymph Node
Thoracic Lymph Node
17010
17020
17021
18009
19007
19011
(M)
(F)

Supplementary Figure 6. *Long-term engraftment of human PLC-mcoET3 in multiple tissue after IUTx*. a) RT-qPCR was performed in triplicate using mcoET3-specific primers on RNA isolated from a) liver, lung, thymus spleen, reproductive organs, mesenteric and thoracic lymph nodes (n=6 animals) and the percentage of PLC-mcoET3 engraftment was extrapolated using a standard curve prepared with RNA isolated from different percentages of PLC-mcoET3 mixed with sheep stromal cells; b-h) percentage of PLC-mcoET3 engraftment in each animal is depicted, and all animals continued to have expression of mcoET3 in all organs tested at different levels. Samples were not available for animals: 17021 thymus, 19011 reproductive tissue, and 18007,19009 mesenteric lymph node. Data are shown as Mean ± SEM. One-way ANOVA followed by Tukey's multiple comparison test was used to determine significant differences and $p \leq 0.05$ was considered significant. (ns >0.05) and ns p values are detailed in sequence from left to right: and ns p values are detailed in sequence from left to right: >0.9999; >0.9999; 0.4465; >0.9999; >0.9999; 0.9980; 0.9999; 0.2980; >0.9999; 0.9999; 0.5702; >0.9999; 0.9996; 0.9947; 0.5247; 0.3685; 0.1547; 0.9999; 0.9971; >0.9999.

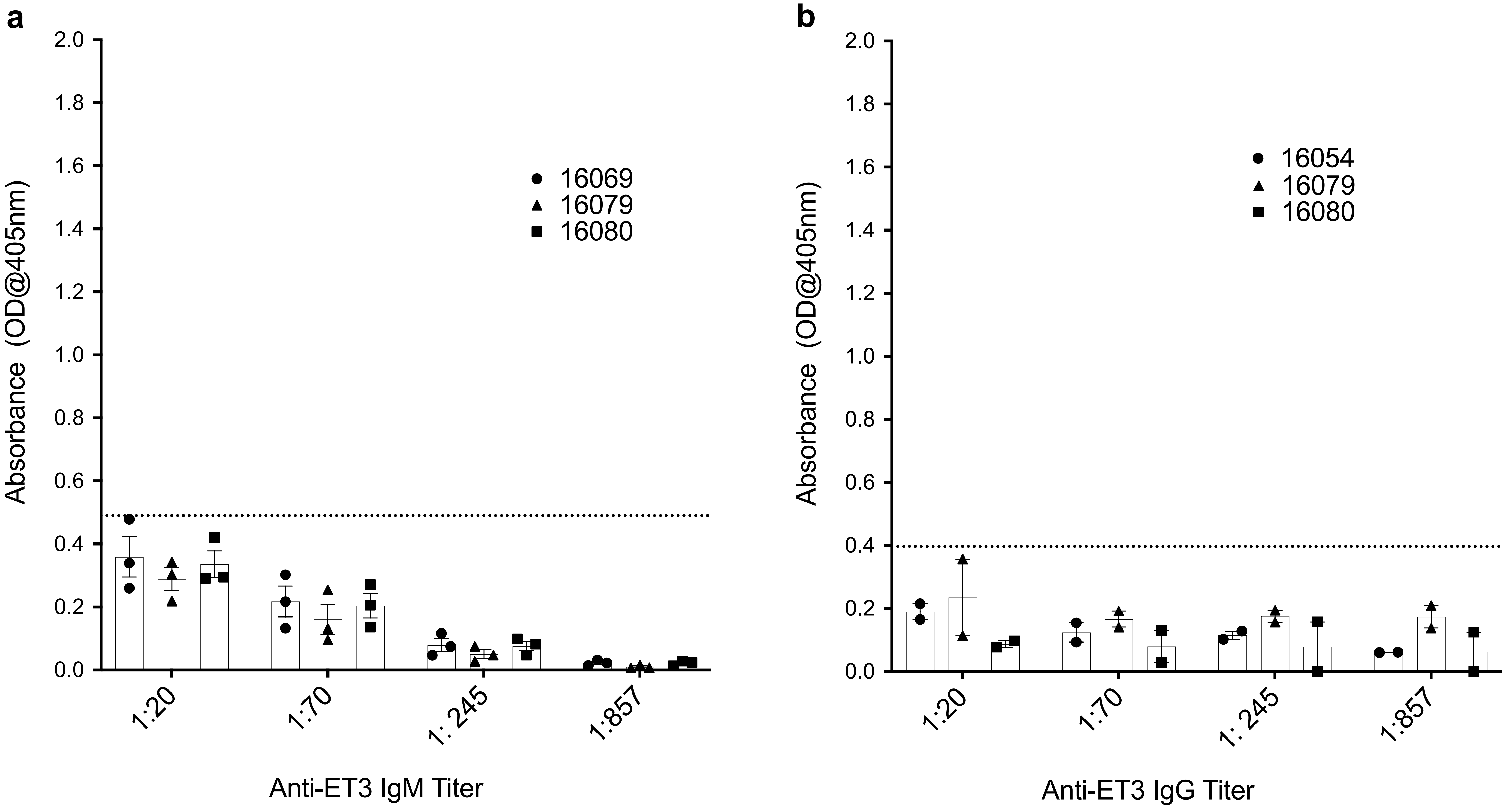
a
b
Absorbance (OD@405nm)
2.0
1.8
1.6
1.4
1.2
1.0
0.8
0.6
0.4
0.2
0.0
16069
16079
16080
16054
1:20
1:70
1: 245
1:857
Anti-ET3 IgM Titer
Anti-ET3 IgG Titer

Supplementary Figure 7. *Evaluation of control non-IUTx sheep for* Anti-ET3 *IgM and IgG.* To determine the threshold for a positive signal in the anti-ET3 specific ELISAs a) IgM and b) IgG ELISAs were performed in triplicate on serial dilutions of plasma from non-IUTx control animals (n=3 animals presented as individual data). Positive antibody titers were defined as the dilution of plasma with an absorbance value >2 SD above the mean OD (dotted line). Data are shown as mean± SEM.
