# Supplementary material for: Transplanting FVIII/ET3-secreting cells in fetal sheep increases FVIII levels long-term without inducing immunity or toxicity
Source: Nat Commun. 2023 Jul 14;14:4206. doi: 10.1038/s41467-023-39986-1 (PMC10349136; doi:10.1038/s41467-023-39986-1)
Supplement: Supplementary file 3 — Reporting Summary [file 41467_2023_39986_MOESM3_ESM.pdf]

nature portfolio

Corresponding author(s): Graca Almeida-Porada

Last updated by author(s): Apr 28, 2023

Reporting Summary

Nature Portfolio wishes to improve the reproducibility of the work that we publish. This form provides structure for consistency and transparency in reporting. For further information on Nature Portfolio policies, see our Editorial Policies and the Editorial Policy Checklist.

Statistics

For all statistical analyses, confirm that the following items are present in the figure legend, table legend, main text, or Methods section.

n/a	Confirmed	
☐	☒	The exact sample size (*n*) for each experimental group/condition, given as a discrete number and unit of measurement
☐	☒	A statement on whether measurements were taken from distinct samples or whether the same sample was measured repeatedly
☐	☒	The statistical test(s) used AND whether they are one- or two-sided<br>*Only common tests should be described solely by name; describe more complex techniques in the Methods section.*
☒	☐	A description of all covariates tested
☐	☒	A description of any assumptions or corrections, such as tests of normality and adjustment for multiple comparisons
☐	☒	A full description of the statistical parameters including central tendency (e.g. means) or other basic estimates (e.g. regression coefficient) AND variation (e.g. standard deviation) or associated estimates of uncertainty (e.g. confidence intervals)
☐	☒	For null hypothesis testing, the test statistic (e.g. F, t, r) with confidence intervals, effect sizes, degrees of freedom and P value noted<br>*Give P values as exact values whenever suitable.*
☒	☐	For Bayesian analysis, information on the choice of priors and Markov chain Monte Carlo settings
☒	☐	For hierarchical and complex designs, identification of the appropriate level for tests and full reporting of outcomes
☒	☐	Estimates of effect sizes (e.g. Cohen's *d*, Pearson's *r*), indicating how they were calculated

Our web collection on statistics for biologists contains articles on many of the points above.

Software and code

Policy information about availability of computer code

Data collection	NA
Data analysis	Prism 9.5.1<br>Fiji/ImageJ Macro (.ijm format): Custom macros were created in the .ijm format to automate a series of tasks, such as normalization, processing, and analysis of a large number of images. The tasks performed by these custom macros include color deconvolution, sharpening, contrast leveling, background subtraction, auto thresholding, watershed function, and particle analysis. The macros were designed for use with the specific version of Fiji/ImageJ mentioned above (2.9.0/1.53t) and Java 1.8.0. The provided code is a custom macro written for Fiji/ImageJ and consists of a series of built-in functions and commands from the ImageJ software. Since ImageJ is in the public domain and Fiji is distributed under the GNU General Public License, the code itself does not have any individual licenses attached to it. The functions used in the macro are part of the core ImageJ and Fiji functionalities, and therefore, no additional licenses apply to the code.<br>Please note that the custom macros we developed are specific to our process and might not be directly applicable to other projects without modification. To reproduce our analysis, access to our custom macros or the creation of a new macro based on the provided description would be required. https://github.com/jhd8593/Martin_IHC.git<br>The ET3 sequence was added to the Ovis aries UP000002356 FASTA, converted to a Prosit compatible CSV format and submitted to Prosit. The converted Prosit file was then converted to the EncyclopeDIA DLIB format. ScaffoldDIA (Proteome Software, Portland, OR) using the Ovis aries UP000002356 DLIB produced an empirically corrected library using the GPF-DIA injections. DIA-MS samples were analyzed using Scaffold DIA (3.1.0). DIA-MS data files were converted to mzML format using ProteoWizard (3.0.19254)<br>Peptide quantification was performed by Encyclopedia (1.2.2)

For manuscripts utilizing custom algorithms or software that are central to the research but not yet described in published literature, software must be made available to editors and reviewers. We strongly encourage code deposition in a community repository (e.g. GitHub). See the Nature Portfolio guidelines for submitting code & software for further information.

Data

Policy information about availability of data

All manuscripts must include a data availability statement. This statement should provide the following information, where applicable:

- Accession codes, unique identifiers, or web links for publicly available datasets
- A description of any restrictions on data availability
- For clinical datasets or third party data, please ensure that the statement adheres to our policy

Provide your data availability statement here.

Research involving human participants, their data, or biological material

Policy information about studies with human participants or human data. See also policy information about sex, gender (identity/presentation), and sexual orientation and race, ethnicity and racism.

Reporting on sex and gender	Placental tissue derived from pregnancies with male fetuses
Reporting on race, ethnicity, or other socially relevant groupings	Information not collected
Population characteristics	Discarded placental tissue
Recruitment	Discarded placentas are obtained after elective cesarean and vaginal deliveries at Atrium Wake Forest Baptist Hospitals in Winston-Salem and High Point from live, term births using WFIRM's Placental Procurement kit with appropriate labeling. Written informed consent is obtained by a research nurse or placenta donation coordinator from each subject using the IRB approved consent form. Laboring subjects are approached prior to delivery in their triage or labor room. Care is taken by the research member to select patients who are managing their pain and are not under duress. The research team member asks the patient if they would prefer to discuss the study and medical history privately, or if their family members may remain in the room. Informed consent is obtained by a member of the research team after a thorough description of the study is given and any questions have been answered. A copy of the signed informed consent document is given to the subject. Compensation for their time according to guidelines from the Office of Human Research Protection and approved by the Wake Forest School of Medicine Institutional Review Board (IRB) is provided.
Ethics oversight	Wake Forest School of Medicine Institutional Review Board (IRB) IRB#00056941

Note that full information on the approval of the study protocol must also be provided in the manuscript.

Field-specific reporting

Please select the one below that is the best fit for your research. If you are not sure, read the appropriate sections before making your selection.

☒ Life sciences ☐ Behavioural & social sciences ☐ Ecological, evolutionary & environmental sciences

For a reference copy of the document with all sections, see nature.com/documents/nr-reporting-summary-flat.pdf

Life sciences study design

All studies must disclose on these points even when the disclosure is negative.

Sample size	Animal numbers were determined by power analyses using G*Power v3.1.9.2, which indicated a minimal # of 4 animals per group.
Data exclusions	No data were excluded from the analysis
Replication	Efficacy of treatment was replicated by the number of animals evaluated in this study over a period of 3 years and the multiple samples that were collected and analyzed at different time points. All tests, such as ELISAs and molecular analyses, were run in triplicate for each test and each time point. IHC was performed on at least 3 slides for each tissue and in 3 different/separate experiments.
Randomization	The goal of this study was to determine whether human placental cells bioengineered to produce an optimized FVIII protein, administered prenatally, resulted in elevation in plasma FVIII levels and for how long. As such we had one treatment group and control untreated animals.
Blinding	When evaluating treatment efficacy, tests such FVIII activity were run blind to animal treatment status, as samples were sent to a clinical lab. In addition, we outsourced analysis from third party independent labs (LC-MS, HLA antibodies, Liver enzymes, hemograms, NGS, or pathologists (tumorigenicity)) to avoid possible bias. Since there was only 1 treatment group and some of the tests required the knowledge of which ones were the control (MLR, ELISA), the operators were required to know which samples were controls, but they were blinded to the ID# of the treated animals being analyzed.

Reporting for specific materials, systems and methods

We require information from authors about some types of materials, experimental systems and methods used in many studies. Here, indicate whether each material, system or method listed is relevant to your study. If you are not sure if a list item applies to your research, read the appropriate section before selecting a response.

Materials & experimental systems

n/a	Involved in the study
☐	☒ Antibodies
☒	☐ Eukaryotic cell lines
☒	☐ Palaeontology and archaeology
☐	☒ Animals and other organisms
☒	☐ Clinical data
☒	☐ Dual use research of concern
☒	☐ Plants

Methods

n/a	Involved in the study
☐	☐ ChIP-seq
☐	☐ Flow cytometry
☐	☐ MRI-based neuroimaging

Antibodies

Antibodies used: Please see detailed information in the Materials and Methods section and the Excel spreadsheet in the Supplementary Materials describing all information regarding the antibodies used.

Validation: Please see information in the Excel spreadsheet in the Supplementary Materials describing all information regarding antibodies used, including the Cat#, the clone, the dilutions used, and the means of validation.

Animals and other research organisms

Policy information about studies involving animals; ARRIVE guidelines recommended for reporting animal research, and Sex and Gender in Research

Laboratory animals: All experimental sheep are an outbred mix of Alpine White, Suffolk, Rambouillet and Merino strains

Wild animals: N/A

Reporting on sex: Fetal sheep (males and females), were injected with PLC-mcoET3 intraperitoneally at 59-65 g.d. The sex of the animal being injected was unknown at the time of injection. Although HA is an X-linked disorder and predominantly affects males, here the product was administered to both males and females. Detailed sex information is provided in Supplementary Table 1.

Field-collected samples: N/A

Ethics oversight: Animal studies were approved by the Wake Forest University School of Medicine Institutional Animal Care and Use Committee (IACUC); Protocol # A22-103.

Note that full information on the approval of the study protocol must also be provided in the manuscript.

Plants

Seed stocks: *Report on the source of all seed stocks or other plant material used. If applicable, state the seed stock centre and catalogue number. If plant specimens were collected from the field, describe the collection location, date and sampling procedures.*

Novel plant genotypes: *Describe the methods by which all novel plant genotypes were produced. This includes those generated by transgenic approaches, gene editing, chemical/radiation-based mutagenesis and hybridization. For transgenic lines, describe the transformation method, the number of independent lines analyzed and the generation upon which experiments were performed. For gene-edited lines, describe the editor used, the endogenous sequence targeted for editing, the targeting guide RNA sequence (if applicable) and how the editor was applied.*

Authentication: *Describe any authentication procedures for each seed stock used or novel genotype generated. Describe any experiments used to assess the effect of a mutation and, where applicable, how potential secondary effects (e.g. second site T-DNA insertions, mosiacism, off-target gene editing) were examined.*

ChIP-seq

Data deposition

- [ ] Confirm that both raw and final processed data have been deposited in a public database such as GEO.
- [ ] Confirm that you have deposited or provided access to graph files (e.g. BED files) for the called peaks.

Data access links<br>*May remain private before publication.*	*For "Initial submission" or "Revised version" documents, provide reviewer access links. For your "Final submission" document, provide a link to the deposited data.*
Files in database submission	*Provide a list of all files available in the database submission.*
Genome browser session<br>(e.g. UCSC)	*Provide a link to an anonymized genome browser session for "Initial submission" and "Revised version" documents only, to enable peer review. Write "no longer applicable" for "Final submission" documents.*

Methodology

Replicates	*Describe the experimental replicates, specifying number, type and replicate agreement.*
Sequencing depth	*Describe the sequencing depth for each experiment, providing the total number of reads, uniquely mapped reads, length of reads and whether they were paired- or single-end.*
Antibodies	*Describe the antibodies used for the ChIP-seq experiments; as applicable, provide supplier name, catalog number, clone name, and lot number.*
Peak calling parameters	*Specify the command line program and parameters used for read mapping and peak calling, including the ChIP, control and index files used.*
Data quality	*Describe the methods used to ensure data quality in full detail, including how many peaks are at FDR 5% and above 5-fold enrichment.*
Software	*Describe the software used to collect and analyze the ChIP-seq data. For custom code that has been deposited into a community repository, provide accession details.*

Flow Cytometry

Plots

Confirm that:

- [ ] The axis labels state the marker and fluorochrome used (e.g. CD4-FITC).
- [ ] The axis scales are clearly visible. Include numbers along axes only for bottom left plot of group (a 'group' is an analysis of identical markers).
- [ ] All plots are contour plots with outliers or pseudocolor plots.
- [ ] A numerical value for number of cells or percentage (with statistics) is provided.

Methodology

Sample preparation	*Describe the sample preparation, detailing the biological source of the cells and any tissue processing steps used.*
Instrument	*Identify the instrument used for data collection, specifying make and model number.*
Software	*Describe the software used to collect and analyze the flow cytometry data. For custom code that has been deposited into a community repository, provide accession details.*
Cell population abundance	*Describe the abundance of the relevant cell populations within post-sort fractions, providing details on the purity of the samples and how it was determined.*
Gating strategy	*Describe the gating strategy used for all relevant experiments, specifying the preliminary FSC/SSC gates of the starting cell population, indicating where boundaries between "positive" and "negative" staining cell populations are defined.*

- [ ] Tick this box to confirm that a figure exemplifying the gating strategy is provided in the Supplementary Information.

Magnetic resonance imaging

Experimental design

Design type	*Indicate task or resting state; event-related or block design.*

Design specifications	*Specify the number of blocks, trials or experimental units per session and/or subject, and specify the length of each trial or block (if trials are blocked) and interval between trials.*
Behavioral performance measures	*State number and/or type of variables recorded (e.g. correct button press, response time) and what statistics were used to establish that the subjects were performing the task as expected (e.g. mean, range, and/or standard deviation across subjects).*

Acquisition

Imaging type(s)	*Specify: functional, structural, diffusion, perfusion.*
Field strength	*Specify in Tesla*
Sequence & imaging parameters	*Specify the pulse sequence type (gradient echo, spin echo, etc.), imaging type (EPI, spiral, etc.), field of view, matrix size, slice thickness, orientation and TE/TR/flip angle.*
Area of acquisition	*State whether a whole brain scan was used OR define the area of acquisition, describing how the region was determined.*

Diffusion MRI ☐ Used ☐ Not used

Preprocessing

Preprocessing software	*Provide detail on software version and revision number and on specific parameters (model/functions, brain extraction, segmentation, smoothing kernel size, etc.).*
Normalization	*If data were normalized/standardized, describe the approach(es): specify linear or non-linear and define image types used for transformation OR indicate that data were not normalized and explain rationale for lack of normalization.*
Normalization template	*Describe the template used for normalization/transformation, specifying subject space or group standardized space (e.g. original Talairach, MNI305, ICBM152) OR indicate that the data were not normalized.*
Noise and artifact removal	*Describe your procedure(s) for artifact and structured noise removal, specifying motion parameters, tissue signals and physiological signals (heart rate, respiration).*
Volume censoring	*Define your software and/or method and criteria for volume censoring, and state the extent of such censoring.*

Statistical modeling & inference

Model type and settings	*Specify type (mass univariate, multivariate, RSA, predictive, etc.) and describe essential details of the model at the first and second levels (e.g. fixed, random or mixed effects; drift or auto-correlation).*
Effect(s) tested	*Define precise effect in terms of the task or stimulus conditions instead of psychological concepts and indicate whether ANOVA or factorial designs were used.*

Specify type of analysis: ☐ Whole brain ☐ ROI-based ☐ Both

Statistic type for inference<br>(See Eklund et al. 2016)	*Specify voxel-wise or cluster-wise and report all relevant parameters for cluster-wise methods.*
Correction	*Describe the type of correction and how it is obtained for multiple comparisons (e.g. FWE, FDR, permutation or Monte Carlo).*

Models & analysis

n/a	Involved in the study
☐	☐ Functional and/or effective connectivity
☐	☐ Graph analysis
☐	☐ Multivariate modeling or predictive analysis

Functional and/or effective connectivity	*Report the measures of dependence used and the model details (e.g. Pearson correlation, partial correlation, mutual information).*
Graph analysis	*Report the dependent variable and connectivity measure, specifying weighted graph or binarized graph, subject- or group-level, and the global and/or node summaries used (e.g. clustering coefficient, efficiency, etc.).*
Multivariate modeling and predictive analysis	*Specify independent variables, features extraction and dimension reduction, model, training and evaluation metrics.*
